# Long-term survival following medical management of *Aspergillus* endocarditis with dissemination as a consequence of steroid therapy in severe COVID-19 pneumonia

**DOI:** 10.1016/j.mmcr.2024.100638

**Published:** 2024-03-01

**Authors:** Kiran G. Kulirankal, Ann Mary, Merlin Moni, Gopal S. Pillai, Dipu T. Sathyapalan

**Affiliations:** a Division of Infectious Diseases, Department of Internal Medicine, Amrita Institute of Medical Sciences, Kochi, India; b Department of Internal Medicine, Amrita Institute of Medical Sciences, Kochi, India; c Department of Ophthalmology, Amrita Institute of Medical Sciences, Kochi, India

**Keywords:** COVID-19, Covid pneumonia, Infectious diseases, Aspergillus fumigatus, Fungal endocarditis, Disseminated Aspergillus

## Abstract

A male in his 40's with no known comorbidities developed severe COVID-19 pneumonia and received a four-week course of methylprednisolone. The patient subsequently developed disseminated *Aspergillus* endocarditis, manifesting as multiple organ involvement including the heart, eyes, and brain. Despite the poor prognosis generally associated with fungal endocarditis, the patient survived following aggressive medical management with a combination of liposomal amphotericin b and voriconazole therapy and is now doing well for over two years and is off antifungal therapy for a year.

## 1 Introduction

In the aftermath of the COVID-19 pandemic, an increase in opportunistic fungal infections has been observed, posing significant challenges to healthcare systems. While invasive aspergillosis primarily affects immunocompromised individuals, rare cases involving immunocompetent patients have been reported.

Specific and universally accepted treatment protocols for COVID-19 were lacking during the early stages of the pandemic. The earliest breakthrough in the management of severe COVID-19 was the use of corticosteroids, particularly dexamethasone. Prolonged usage of steroids, particularly in higher doses, can have immunosuppressive effects further increasing the risk of infections. COVID-19 associated pulmonary aspergillosis (CAPA) is a serious complication leading to increased mortality and morbidity [1]. Fungal infections, including *Aspergillus* endocarditis, have emerged as significant complications in patients with severe COVID-19 pneumonia who receive steroid therapy [2].

This case report documents the successful medical treatment of a rare and difficult case of disseminated invasive aspergillosis including endocarditis in a patient who received intravenous methylprednisolone for severe COVID 19 pneumonia. The favorable outcome of this case offers valuable insights into the potential for effective treatment of this life-threatening infection and serves as a significant addition to the existing literature.

## 2 Case presentation

A male in his early 40's with no known co-morbidities presented to an external hospital with complaints of fever, rhinitis, and cough during the second wave of COVID-19 (end of 2021). He was diagnosed with COVID-19 by an RT-PCR test. Initially experiencing mild symptoms, his condition worsened after 2 weeks, requiring nasal oxygen, antiviral medication (intravenous remdesivir), antibiotics, and non-invasive ventilation. He was initiated on intravenous steroids (methylprednisolone) in view of worsening pneumonia. With the above measures he improved and was discharged after a month.

Two days post-discharge, the patient developed acute onset left-sided chest pain and breathlessness (day 0). An X-ray chest at a local hospital revealed a left-sided pneumothorax, which was stabilized with the insertion of an intercoastal chest drain. During his stay, he experienced pain and blurring of vision of his left eye, followed by the right eye (day 6). Ophthalmological examination suggested bilateral endophthalmitis, and the patient was referred to our center, a tertiary care referral hospital in south India for expert management.

**Fig. 1:**
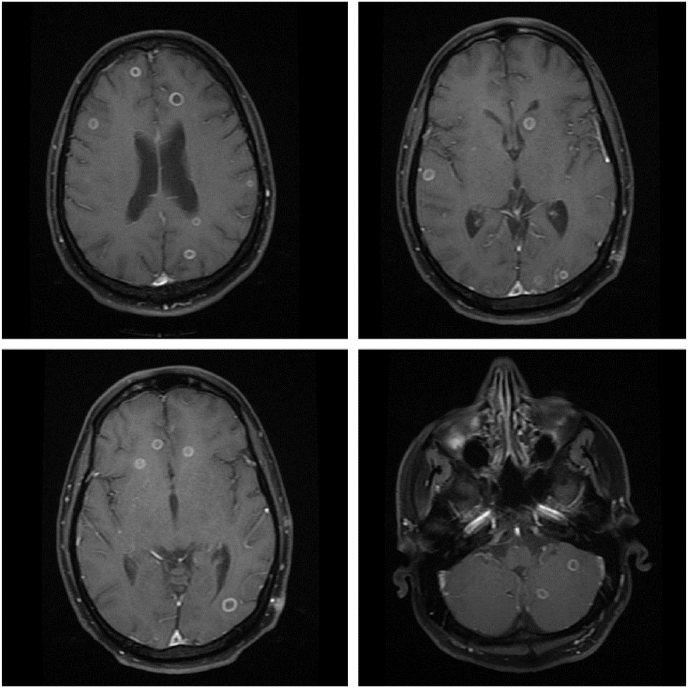
Axial T1 weighted sequence showing multiple ring enhancing lesions scattered throughout the brain parenchyma.

In the emergency room (day 7), the patient's Glasgow coma scale (GCS) was full, and he maintained saturation of 97% with 2 L of oxygen. Ophthalmology consultation confirmed bilateral endophthalmitis. Blood, urine, and vitreous cultures were collected, and the patient was started empirically on intravenous meropenem (1 gm IV q8h) and intravenous fluconazole (200mg IV q24h) after admission to the medical intensive care unit. Initial blood cultures grew gram-positive cocci (day 9), and the patient was started on intravenous teicoplanin (400mg q12h x 3 doses followed by 400mg IV q24h) for gram-positive coverage and later discontinued as it turned out to be coagulase negative staphylococci. Magnetic resonance imaging (MRI) of the brain revealed multiple ring-enhancing lesions (Fig. 1), while computed tomography (CT) of the thorax showed ground glass opacity and fibrotic bands suggestive of post-COVID lung changes and a resolved pneumothorax.

**Fig. 2:**
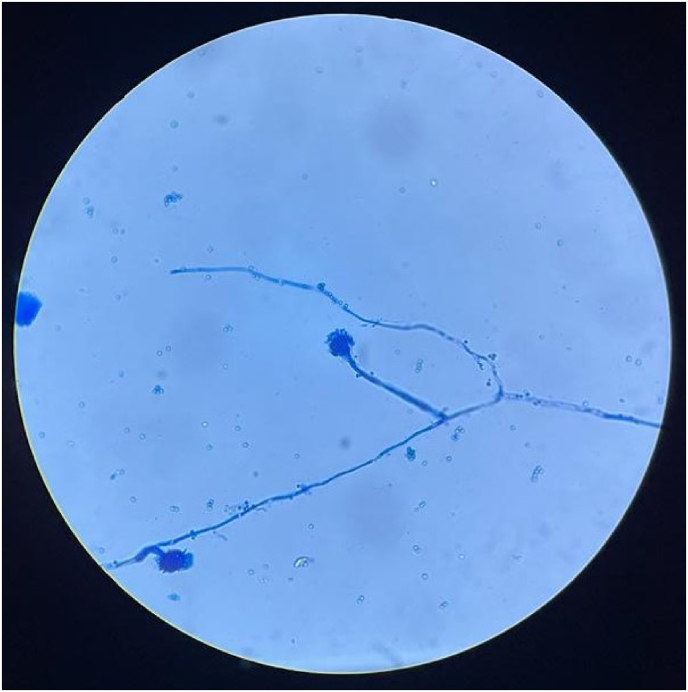
Lacto-Phenol-Cotton-Blue mount from vitral culture showing hyaline septate hyphae suggestive of *Aspergillus fumigatus*. (For interpretation of the references to colour in this figure legend, the reader is referred to the Web version of this article.)

Vitreous smear showed (day 10) septate narrow hyphae, suggestive of *Aspergillus* spp. (Fig. 2). The patient was started on intravenous voriconazole (400mg IV q12h x 2 doses followed by 200mg q12h) after stopping fluconazole. Five days later, the vitreous culture from the left eye grew *Aspergillus fumigatus*. Induced sputum cultures also grew *Aspergillus* species, and serum galactomannan was 3.4 (>0.5: positive).

**Fig. 3:**
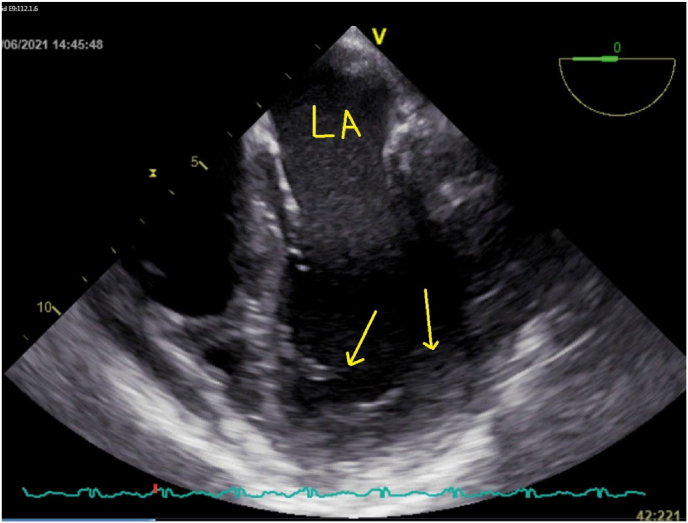
2-dimensional echocardiogram showing filamentous fibrinous structural along pericardial floating into the fluid.

A 2-dimensional cardial echo scan revealed vegetations attached to the heart, with transesophageal echocardiogram (TEE) showing extensive mural vegetations in all cardiac chambers, thickening of ventricular walls, mild pericardial effusion, and filamentous fibrinous structures in the pericardial fluid (Fig. 3).

**Fig. 4:**
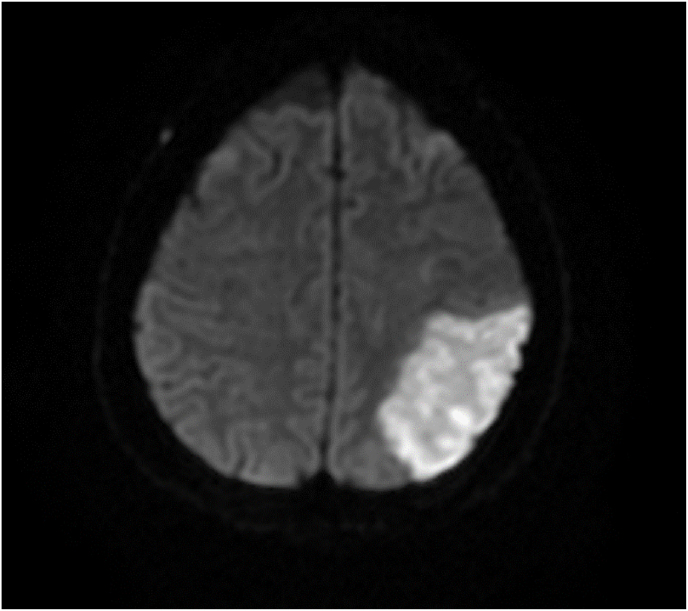
MRI-cerebrum:Axial diffusion weighted image showing acute left middle cerebral artery infarct.

The patient developed sudden anomic aphasia and decreased motor power in the right upper limb (day 28), and MRI revealed left middle cerebral artery (MCA) infarct (Fig. 4). Stroke medicine opinion was obtained, and patient was started on subcutaneous enoxaparin 0.4ml q24h. In view of multiple septic embolization, a carotid artery doppler was done (day 32), which showed no flow-limiting plaque/stenosis. Surgical source control was deemed limited in utility by the cardiothoracic team due to extensive mural vegetations involving all four chambers. Multidisciplinary team discussions were held, and it was decided to administer systemic and intra-vitreal antifungal therapy, using dual voriconazole (systemic dose: 400mg IV q12h x 2 doses followed by 200mg q12h, intra-vitreal dose:100 microg/0.1 ml) and liposomal amphotericin B (systemic dose: 300mg IV q24h, intra-vitreal dose: 5 μg/0.1 mL).

The patient's general condition improved, with aphasia resolving and inflammatory markers improving, and oxygen requirements tapering off. Repeat MRI brain after 6 weeks showed a significant decrease in the size of the ring-enhancing lesions and perilesional oedema. However, the patient's left eye vision continued to worsen, leading to vitrectomy, silicone oil injection, and eventual evisceration due to progressive pan-ophthalmitis (day 52). Despite worsening vision, the right eye intravitreal liposomal amphotericin B injections were continued, along with parasplana vitrectomy, endolaser, fluid-air exchange, membrane peeling, and silicone oil injections which led to salvage of the vision in the right eye.

Liposomal amphotericin B was discontinued after 90 days (day 125), and the patient was maintained on oral voriconazole with adequate levels (between 1.8 and 4.2ug/ml).

A positron emission tomography (PET) CT scan (day 128) indicated residual infection in the right orbit, while a follow-up echo at three months revealed good LV systolic function with no vegetations. The patient received a left orbital prosthesis (day 131), underwent ocular rehabilitation and the visual acuity of the right eye gradually improved to 6/60 over the next three months. After a four-month hospital stay, he was discharged (day 152) on oral voriconazole (200mg q12h) treatment for 1 year in total. The patient was reviewed every 3–4 months and remained asymptomatic. At the time of writing this review, the patient has been off antifungals for an additional year without any recurrence.

## 3 Discussion

Although our patient's CT thorax scan showed ground glass opacity and fibrotic bands suggestive of post COVID-19 lung changes with complete resolution of pneumothorax, sputum culture grew *Aspergillus.* The development of COVID-19 associated pulmonary aspergillosis (CAPA) remains poorly understood but is believed to be linked to structural lung damage and impaired immune response due to COVID-19 infection [3]. The most important risk factors include severe lung damage during the course of COVID-19, the use of corticosteroids in those with ARDS, the widespread use of broad-spectrum antibiotics in intensive care units, and the presence of comorbidities such as structural lung defects [3].The release of danger-associated molecular patterns (DAMPs), signal molecules released by dying or damaged cells that act as endogenous danger signals to promote and exacerbate the immune and inflammatory response leads to lung injury [3].The diagnostic algorithm of CAPA remains difficult as BAL fluid galactomannan testing and culture are not routinely done in patients with COVID-19 due to risk of disease transmission.

Endogenous fungal endophthalmitis is a sight threatening intra ocular infection occurring because of hematogenous spread of infection from a remote systemic location [4]. Our patient developed bilateral endophthalmitis as part of disseminated aspergillosis post severe COVID-19 pneumonia. Early detection of eye involvement and treatment are crucial for preserving vision [5]. Severe fungal infections have been noted in COVID-19 patients, particularly during the middle and latter stages of the disease [6]. The increasing trend of fungal endophthalmitis post COVID-19 presents challenges due to its subacute course, delayed presentation, and limited availability of effective antifungal drugs with good ocular penetration [7]. The poor prognosis and difficulty in salvaging the eye has been exemplified in our case as the aggressive systemic therapy and intraocular injections were able to salvage only one eye.

The diagnosis of disseminated invasive aspergillosis in our patient was based on culture-positive *Aspergillus* from intra-vitreous and sputum cultures, and a positive galactomannan test, and suggestive radiological abnormalities on the MRI brain and echocardiogram. The diagnosis of fungal endocarditis, in our patient, an infrequent but debilitating condition with a poor prognosis, could explain the extensive dissemination observed. Invasive aspergillosis has a mortality ranging from 40% to 95% [8]. Prognosis in patients with *Aspergillus* endocarditis is poor, with an estimated 96% mortality rate in patients treated with medical therapy alone [9]. Surgically treated patients had a lower inpatient mortality when compared to those treated medically [10]. Early diagnosis is crucial for appropriate antimicrobial therapy and surgical consideration. It is not well-known what percentage of patients with *Aspergillus* endocarditis have isolated mural involvement, but it has been reported to be up to 41% in autopsy reports [11]. The extensive mural involvement and involvement of all the four chambers made it practically impossible to consider surgical therapy and had to proceed with the combination antifungal therapy.

Cure of Aspergillus endocarditis without surgical valve replacement is rare. Even with aggressive medical and surgical treatment, survival rates have been reported to be <20% [12]. The mortality of medically managed cases without surgery approaches 100% [13]. One remarkable highlight of our case is that the patient had a favorable outcome without surgery and improved with medical management alone which is rarely reported.

This case report underscores the need for early detection and aggressive treatment of fungal infections in patients recovering from severe COVID-19 pneumonia and treated with immunosuppressive agents, to improve clinical outcomes. Despite the near 100% mortality rate typically associated with disseminated aspergillosis including the lungs, heart, brain and eyes, the patient survived (albeit with losing his left eye), due to timely intervention, comprehensive medical management, and the reversible nature of the immunosuppression he has experienced.

## CRediT authorship contribution statement

**Kiran G. Kulirankal:** Conceptualization, Writing – review & editing. **Ann Mary:** Methodology, Patient treatment. **Merlin Moni:** Writing, Supervision. **Gopal S. Pillai:** Patient treatment. **Dipu T. Sathyapalan:** Writing – original draft, preparation.

## Declaration of competing interest

There are none.
